# Impact of Cirrhosis Etiology and Intensive Care Accessibility on Upper Gastrointestinal Bleeding: A Prospective Observational Study

**DOI:** 10.1155/cjgh/1409025

**Published:** 2026-05-30

**Authors:** Mariana Barros Marcondes, Clara Fantinelli Moreno, Cíntia Mitsue Pereira Suzuki, Maxwell Antonio Garcia Rodrigues, Alecsandro Moreira, Xingshun Qi, Fernando Gomes Romeiro

**Affiliations:** ^1^ Department of Internal Medicine, São Paulo State University (UNESP), Botucatu, São Paulo, Brazil, unesp.br; ^2^ Department of Gastroenterology, General Hospital of Northern Theater Command, Shenyang, China, syjqzyy.com

**Keywords:** alcohol abstinence, cirrhosis, mortality, portal hypertension, upper gastrointestinal bleeding

## Abstract

**Background:**

Upper gastrointestinal bleeding (UGIB) is a significant cause of cirrhosis decompensation; however, there is still controversy on risk factors for poor outcomes. This study aims to explore the impact of additional variables, such as liver disease etiology and intensive care unit (ICU) accessibility, on mortality, rebleeding, and infection rates.

**Methods:**

Cox regression analysis was employed to identify predictors associated with mortality and rebleeding, while Poisson regression analysis was utilized to assess associations with infections.

**Results:**

A total of 228 patients were included, with the majority classified as Child‐Pugh B and C. Among them, 96 patients (42.1%) had alcohol‐associated liver disease (ALD), of which 45 (46.9%) were in alcohol abstinence. One hundred and ninety‐five patients (85.5%) survived, while 33 (14.5%) died. Intubation was required for 31 patients (13.6%), and 28 were transferred to the ICU. Antibiotic prophylaxis was administered to 219 patients (96%), and 55 (24.1%) developed infections. Both orotracheal intubation and transfusions were associated with high mortality, whereas ALD was linked to a lower risk of death (*p* < 0.001, 0.029, and 0.005, respectively). Intubation was also correlated with an increased risk of rebleeding, while transfer to ICU reduced this complication (*p* = 0.012 and 0.045, respectively). The Child‐Pugh score and length of hospitalization were associated with the occurrence of infections (*p* = 0.037 and < 0.001, respectively).

**Conclusions:**

Intubation, transfusions, liver disease etiology, and ICU accessibility are critical predictors following UGIB in cirrhosis. Additionally, the Child‐Pugh score and duration of hospitalization are directly proportional to the risk of infections in this context.

**Trial Registration:** ClinicalTrials.gov identifier: NCT04662918

## 1. Introduction

Cirrhosis is a global health burden that predominantly affects individuals aged 25–49 years [1, 2]. Despite advances in the management of hepatitis B and C, alcohol‐associated liver disease (ALD) is the leading cause of cirrhosis in Europe and the Americas [2]. While compensated cirrhosis is associated with favorable outcomes, decompensation significantly worsens prognosis. In this stage, systemic inflammation and portal hypertension drive disease progression, and mortality risk can be estimated by integrating inflammatory markers with portal hypertension parameters [3].

Upper gastrointestinal bleeding (UGIB) is among the most frequent complications of decompensated cirrhosis, occurring in 25%–40% of patients and leading to mortality rates between 20% and 30% [1, 4]. In this context, poor outcomes are linked to advanced liver disease and failure to achieve primary hemostasis. Additional predictors of adverse prognosis include hepatic encephalopathy (HE), liver failure, sepsis, acute kidney injury (AKI), and rebleeding [5, 6].

Numerous retrospective studies have explored the factors contributing to increased mortality in cirrhotic patients with UGIB. Specific prognostic tools, such as the Cirrhosis Acute Gastrointestinal Bleeding (CAGIB) score, have also been utilized and enhanced through machine learning techniques, demonstrating comparable performance to traditional models in predicting in‐hospital outcomes [7]. Although it is strongly recommended that these patients be managed in intensive or intermediate care settings, the supporting evidence remains limited [8].

The prognostic impact of liver disease etiology remains challenging to assess. A nationwide German study involving 65,357 patients identified ALD as the predominant cause of cirrhosis (60.6%). The overall in‐hospital mortality rate was 18.6%, with worse outcomes associated with hypovolemic shock, sepsis, AKI, mechanical ventilation, acute respiratory distress syndrome, HE, anemia, and male sex. Notably, cirrhosis etiology was not independently linked to prognosis [9]. Conversely, a multicenter prospective study in Italy reported an association between ALD and six‐week mortality [10]. The lack of data on alcohol abstinence in UGIB studies may partly explain these conflicting findings regarding ALD‐related mortality.

Another underexplored factor is access to intensive care units (ICUs), which varies significantly across countries [11]. A retrospective analysis of ICU‐admitted patients revealed that repeated endoscopic interventions and delays in ICU referral were associated with increased rates of rebleeding and mortality [12].

In this prospective study conducted in a country where alcohol‐related liver disease (ALD) is the predominant cause of cirrhosis, we carefully evaluated alcohol abstinence alongside ICU accessibility, liver disease severity, endoscopic findings, and complications occurring during hospitalization, such as the need for mechanical ventilation or esophageal balloon tamponade. The impact of these variables on mortality, rebleeding, and infection rates was assessed. The study hypothesized that alcohol abstinence, clinical complications, and ICU accessibility significantly influence these three endpoints. The novelty of this study lies in evaluating alcohol abstinence and ICU accessibility, and in comparing the impact of these factors—alongside liver disease severity scores and additional clinical variables—on three key outcomes: mortality, rebleeding, and infections.

## 2. Methods

### 2.1. Ethics Approval

The study was approved by the local ethics committee (protocol number 4.462.045–2020) and was in accordance with the 1964 Helsinki declaration and its later amendments or comparable ethical standards.

Patients with cirrhosis and UGIB admitted to the emergency department of a single tertiary center in Brazil between January 2021 and March 2025 were prospectively enrolled following their informed consents. Eligibility criteria included age ≥ 18 years at admission. Patients were excluded if clinical data required to calculate the Child‐Pugh, model for end‐stage liver disease adjusted for serum sodium (MELD‐Sodium), or CAGIB scores were unavailable. This cohort represents the single‐center arm of a prospective, multicenter clinical trial designed to evaluate prognostic scoring systems at hospital admission, titled Validation of the CAGIB score for In‐hospital Mortality of Cirrhotic Patients With Acute Gastrointestinal Bleeding (). Following the multicenter registration, the single‐center arm received independent approval from the institutional review board. Both the multicenter and single‐center components were conceived concurrently, and all data included in the present analysis were prospectively collected. Although the objectives and outcomes were identical in both the multicenter and single‐center studies, the latter included one additional variable—alcohol abstinence—which was not evaluated in the multicenter study.

Epidemiological variables collected included age, sex, and cirrhosis etiology. For patients with ALD, current alcohol consumption or abstinence status was additionally recorded. Data regarding antibiotic prophylaxis, endoscopic findings, and in‐hospital complications were meticulously documented, including the need for balloon tamponade, mechanical ventilation, transfusion requirements, and infectious events. Organ failure was assessed and classified according to the acute‐on‐chronic liver failure (ACLF) criteria [13]. Additional prognostic scores were calculated as follows [14–16]:

 MELD = 3.8 × ln [TB (mg/dL)] + 11.2 × ln [INR] + 9.6 × ln [Cr (mg/dL)] + 6.43.
 MELD‐sodium = MELD + 1.32 × (137−Na) − [0.033 × MELD × (137−Na)]
 CAGIB = diabetes (yes = 1, no = 0) × 1.040 + hepatocellular carcinoma (HCC) (yes = 1, no = 0) × 0.974 + TB (μmol/L) × 0.005 – ALB (g/L) × 0.091 + ALT (U/L) × 0.001 + Cr (μmol/L) × 0.012 – 3.96.
 ALB: albumin, ALT: alanine aminotransferase, Cr: serum creatinine, HCC: hepatocellular carcinoma, INR: international normalized ratio, TB: total bilirubin.

For patients who developed infections, data regarding the infection site, identified microorganisms, and administered antibiotic regimens were collected. Additional clinical variables included the need for mechanical ventilation, esophageal balloon tamponade, and transfer to the ICU.

### 2.2. Statistical Analysis

Cox proportional hazards regression was employed to identify the predictors of mortality and rebleeding. For each outcome, variables were included in the multivariate model if they demonstrated a bivariate association with the dependent variable at a significance level of *p* ≤ 0.20.

Poisson regression analysis was used to evaluate the predictors of infection. As with the other models, variables were selected for multivariate analysis based on a bivariate *p*‐value threshold of ≤ 0.20.

Survival rates among patients with ALD were compared according to alcohol abstinence status using the log‐rank test (Mantel–Cox).

Statistical significance was defined as *p* < 0.05. All analyses were performed using SPSS software, Version 20.0 (IBM Corp., Armonk, NY, USA).

## 3. Results

**FIGURE 1:**
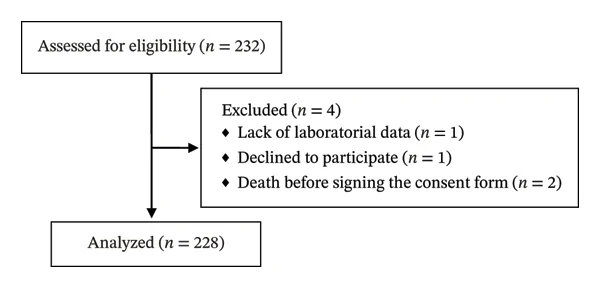
Study flowchart.

A total of 232 patients were screened for eligibility. Four were excluded, resulting in a final cohort of 228 patients included in the analysis (Figure 1). The majority of participants were classified as Child‐Pugh B or C. Of the total sample, 195 patients (85.5%) survived and were discharged, while 33 (14.5%) died during hospitalization.

Regarding ACLF status, 196 patients (86%) had no ACLF at admission. Ten patients (4.4%) were classified as ACLF Grade 1, four (1.7%) as Grade 2, and eighteen (7.9%) as Grade 3. Among those with ACLF Grade 3, only 3 patients (17%) survived, whereas 15 (83%) died.

ALD was the most prevalent etiology, affecting 96 patients (42.1%). Metabolic dysfunction–associated steatotic liver disease (MASLD) was identified in 66 patients (28.9%). The overlap of these two etiologies, referred to as metabolic dysfunction and alcohol‐associated liver disease (MetALD), was present in 18 patients (7.9%). Other etiologies included cryptogenic cirrhosis in 16 patients (7%), hepatitis C in 10 (4.3%), and hepatitis B in 8 (3.5%). Six patients (2.6%) had cirrhosis due to combined ALD and hepatitis C. Autoimmune hepatitis (AIH) was diagnosed in 3 patients (1.3%), one of whom also had MASLD and another had AIH with concomitant primary sclerosing cholangitis (PSC). Two patients (0.9%) had cirrhosis due to ALD and hepatitis B, and two (0.9%) due to MASLD and hepatitis B. One patient (0.4%) had progressive familial intrahepatic cholestasis (PFIC) Type 3.

Table 1 summarizes the baseline characteristics of the study population.

**TABLE 1:** Demographic and clinical data.

Variables	Patients (*n* = 228)
Demographics	
Age (years)	61.0 ± 12.5
Men/women	66.6%/33.3%
Causes of cirrhosis	
ALD	42.1%
MASLD	28.9%
MetALD	7.9%
Viral hepatitis	7.9%
Cryptogenic cirrhosis	7.0%
ALD and hepatitis C	2.6%
HAI	1.3%
ALD and hepatitis B	0.9%
MASLD and hepatitis B	0.9%
PFIC	0.4%
Past medical history	
Previous admission for UGIB	29.82%
HCC	5.2%
Diabetes	46.4%
Laboratory values	
Red blood cells (10^9^/mm^3^)	2.7 (2.29–3.24)
Hemoglobin (g/dL)	8.1 (6.43–9.9)
Hematocrit (%)	24.8 (20.9–29.98)
Liver disease severity scores	
Child‐Pugh A	24.1%
Child‐Pugh B	46.4%
Child‐Pugh C	29.3%
Child‐Pugh (points)	8 (7–10)
CAGIB	−4.62 (−5.26 to −3.69)
MELD	14.76 (10.59–19.46)
MELD‐sodium	14.96 (10.47–21.57)
No ACLF	86%
ACLF 1	4.4%
ACLF 2	1.7%
ACLF 3	7.9%

*Note:* CAGIB, Cirrhosis Acute Gastrointestinal Bleeding; HAI, autoimmune hepatitis; HCC, hepatocellular carcinoma; MASLD, metabolic dysfunction–associated steatotic liver disease; MELD‐sodium, model for end‐stage liver disease adjusted for serum sodium; MetALD, metabolic dysfunction and alcohol‐associated liver disease; UGIB, upper gastrointestinal bleeding.

Abbreviations: ACLF, acute‐on‐chronic liver failure; ALD, alcoholic liver disease; MELD, model for end‐stage liver disease; PFIC, progressive familial intrahepatic cholestasis.

Among the 96 patients with ALD, 45 (46.9%) reported alcohol abstinence, with a mean survival of 199.3 ± 141.8 days. The remaining 51 patients (53.1%) continued consuming alcoholic beverages, and their mean survival was 30.9 ± 5.5 days (*p* = 0.239).

Hematemesis was observed in 80 patients (35.1%), while melena was reported in 55 (24.1%). Both manifestations were present in 70 patients (30.7%). Hematochezia occurred in 17 patients (7.5%), and 6 individuals (2.6%) presented with symptoms of new‐onset anemia.

Ascites was documented in 102 patients (44.7%), with Grade 3 ascites in 49 (21.5%), Grade 2 in 29 (12.7%), and Grade 1 in 24 (10.5%). HE was present in 69 patients (30.3%), with Grade I in 8 (3.5%), Grade II in 34 (14.9%), Grade III in 20 (8.7%), and Grade IV in 7 (3.0%). HCC was diagnosed in 12 patients (5.3%), with half of the cases identified during hospitalization. Additionally, 106 patients (46.5%) had Type 2 diabetes mellitus.

Esophageal balloon tamponade was applied in 8 patients (3.5%), with six procedures conducted prior to endoscopy and two following rebleeding episodes. One patient died from hemorrhagic shock prior to undergoing endoscopy. All remaining participants underwent endoscopic evaluation. In 186 patients (81.6%), UGIB was attributed to variceal hemorrhage, with esophageal varices identified in 170 cases and gastric varices in 16. Other etiologies included peptic ulcers in 15 patients (6.5%), portal hypertensive gastropathy in 15 (6.5%), gastric antral vascular ectasia (GAVE) in 8 (3.5%), and erosive esophagitis in 7 (3.0%). Dieulafoy lesions were found in 3 patients (1.3%), and three others presented with bleeding from band‐induced ulcers at previous ligation sites (1.3%). Single cases were observed for ectopic varices (0.4%), Cameron ulcer (0.4%), and infiltrative malignancy (0.4%). Twelve patients (5.3%) had multiple concurrent sources of bleeding.

Band ligation was performed in 174 cases of esophageal varices, whereas cyanoacrylate injection was used in 16 cases of gastroesophageal or isolated gastric varices. Nine patients underwent sclerotherapy for peptic ulcer bleeding, and 18 received other endoscopic interventions, including hemostatic clipping or argon plasma coagulation.

Splanchnic vasoconstrictors were administered to 200 patients (87.7%), with octreotide used in 198 cases and terlipressin in 2. Antibiotic prophylaxis was provided to 219 participants (96%). Ceftriaxone was the most commonly prescribed antibiotic, administered to 202 patients (88.6%), including 7 who received it in combination with another antibiotic. Four patients (1.7%) received cefepime, while three (1.3%) were treated with other broad‐spectrum agents, such as meropenem or piperacillin/tazobactam. Two patients (0.8%) received dual antibiotic regimens, and one patient (0.4%) was treated with trimethoprim–sulfamethoxazole. Proton pump inhibitors were used in 191 cases, and lactulose was prescribed to 79 patients. Additionally, two patients received L‐ornithine L‐aspartate.

Blood transfusion was not required in 117 individuals (51.3%), whereas 111 patients (48.7%) received packed red blood cells (PRBCs). Among those transfused, the mean number of PRBC units per patient was 1.88 ± 0.98, with a range of 1–5 units. Among those who received PRBCs, additional blood components were administered in selected cases: Five patients received fresh frozen plasma (FFP), one received platelets, and two received cryoprecipitate. Three patients received all three types of blood products. Notably, six of the eight patients who received two or more types of blood components died, corresponding to a mortality rate of 75% within this subgroup. It is worth noting that the patients who received FFP had a median INR of 2.33 (IQR 2.03–3.13) and a median hemoglobin level of 5.5 g/dL (IQR 5.1–6.0).

Orotracheal intubation for mechanical ventilation was performed in 31 patients (13.6%), of whom 21 died, resulting in a mortality rate of 67.7% within this subgroup. Among them, 22 patients (9.6%) were intubated due to altered mental status associated with HE Grades III and IV. In eight cases (3.5%), intubation was indicated for airway protection or in response to cardiorespiratory arrest or epileptic seizures. One patient (0.4%) required intubation due to hemorrhagic shock. Owing to ICU beds shortage, only 28 patients were transferred to the ICU and three intubated patients remained in general medical wards.

The leading cause of death was multiorgan failure, accounting for 29 cases (12.7%). Hemorrhagic shock was responsible for three deaths (1.3%), and one patient (0.4%) died due to infection. Table 2 presents the bivariate Cox regression analysis, showing associations between independent variables and mortality. Table 3 summarizes the multivariate analysis, which identified ALD as independently associated with a lower risk of death. Conversely, the number of PRBC units transfused and the need for intubation were independently associated with increased mortality. The association between ICU transfer and reduced mortality did not reach statistical significance.

**TABLE 2:** Bivariate Cox regression model to evaluate mortality.

Variables	HR	95% confidence interval		*p*
Demographics				
Sex	1.428	0.609	3.351	0.413
Age	1.025	0.995	1.056	0.104
Causes of cirrhosis				
ALD	0.555	0.247	1.244	0.153
MASLD	1.247	0.540	2.879	0.605
MetALD	1.528	0.360	6.496	0.566
Cryptogenic cirrhosis	0.861	0.202	3.664	0.839
Past medical history				
Previous upper gastrointestinal bleeding	1.359	0.615	3.000	0.448
Hepatocellular carcinoma	1.874	0.439	8.006	0.397
Diabetes	1.114	0.525	2.363	0.778
Manifestations of gastrointestinal bleeding				
Hematemesis	1.071	0.498	2.303	0.861
Melena	0.773	0.264	2.266	0.639
Hematemesis and melena	0.997	0.456	2.179	0.995
New‐onset anemia	3.783	0.882	16.226	0.073
Cirrhosis complications at admission				
Ascites	1.174	0.563	2.446	0.669
Hepatic encephalopathy	1.218	0.939	1.580	0.137
Laboratory values				
Hemoglobin	1.000	0.859	1.165	0.995
Hematocrit	0.999	0.948	1.053	0.980
White blood cells	1.000	1.000	1.000	0.330
Neutrophils	1.000	1.000	1.000	0.222
Lymphocytes	1.000	1.000	1.000	0.751
Platelets	1.000	1.000	1.000	0.309
Sodium	0.956	0.897	1.020	0.172
Total proteins	0.723	0.544	0.961	0.026
Blood urea nitrogen	1.022	1.012	1.033	< 0.001
Liver disease severity scores				
Child‐Pugh	1.324	1.123	1.560	0.001
CAGIB	1.370	1.147	1.637	0.001
MELD	1.083	1.039	1.129	< 0.001
MELD‐sodium	1.074	1.032	1.118	< 0.001
ACLF	2.227	1.698	2.920	< 0.001
In‐hospital events				
HCC diagnosed after admission	1.085	0.146	8.078	0.936
ICU admission	4.302	1.945	9.518	< 0.001
Intubation/mechanical ventilation	7.730	3.360	17.783	< 0.001
Balloon tamponade	2.612	0.928	7.352	0.069
Vasoconstrictor	1.853	0.672	5.109	0.233
Lactulose	0.924	0.428	1.992	0.840
Proton pump inhibitor	0.506	0.167	1.533	0.228
Transfusions	1.288	0.613	2.708	0.504
Units of packed red blood cells	1.328	0.990	1.780	0.058
Units of fresh frozen plasma	1.256	1.070	1.474	0.005
Units of platelets	7.413	0.967	56.861	0.054
Units of cryoprecipitate	1.304	1.024	1.661	0.031
Rebleeding	1.062	0.416	2.716	0.900
Endoscopic assessment and therapy				
Esophageal varices	0.624	0.211	1.840	0.392
Gastric varices	0.541	0.163	1.799	0.316
Variceal bleeding	1.410	0.489	4.066	0.525
Peptic ulcer bleeding	0.875	0.207	3.696	0.856
Bleeding due to other causes	0.536	0.162	1.778	0.308
Band ligation	0.891	0.377	2.104	0.792
Cyanoacrylate injection	0.296	0.040	2.202	0.234
Peptic ulcer endoscopic treatment	3.035	0.708	13.006	0.135
Other types of endoscopic treatment	1.421	0.426	4.748	0.568

*Note:* CAGIB, Cirrhosis Acute Gastrointestinal Bleeding score; HCC, hepatocellular carcinoma; MASLD, metabolic dysfunction–associated steatotic liver disease; MetALD, metabolic dysfunction and alcohol‐associated liver disease.

Abbreviations: ACLF, acute‐on‐chronic liver failure; ALD, alcohol‐associated liver disease; HR, hazard ratio; ICU, intensive care unit; MELD, model for end‐stage liver disease.

**TABLE 3:** Multivariate Cox regression model to evaluate mortality.

Variable	HR	95% confidence interval		*p*
Demographics				
Age	1.014	0.967	1.063	0.572
Causes of cirrhosis				
ALD	0.102	0.021	0.493	0.005
Manifestations of gastrointestinal bleeding				
New‐onset anemia	0.450	0.043	4.656	0.503
Cirrhosis complications at admission				
Hepatic encephalopathy	2.360	0.722	7.717	0.155
Laboratory values				
Sodium	0.997	0.847	1.174	0.971
Serum total proteins	0.919	0.533	1.585	0.762
Blood urea nitrogen	1.008	0.991	1.026	0.366
Liver disease severity scores				
Child‐Pugh	0.836	0.628	1.114	0.221
CAGIB	0.838	0.413	1.702	0.626
MELD	1.025	0.776	1.355	0.862
MELD‐sodium	1.110	0.869	1.420	0.403
ACLF	1.427	0.803	2.536	0.226
In‐hospital events				
Balloon tamponade	1.425	0.281	7.236	0.669
Intubation/mechanical ventilation	40.526	5.495	298.902	< 0.001
ICU transference	0.117	0.012	1.193	0.070
Units of packed red blood cells	1.866	1.068	3.261	0.029
Units of fresh frozen plasma	0.833	0.597	1.164	0.285
Endoscopic assessment and therapy				
Peptic ulcer treatment	0.459	0.035	6.097	0.555

*Note:* CAGIB, Cirrhosis Acute Gastrointestinal Bleeding; MELD, model for end‐stage liver disease; MELD‐sodium, MELD adjusted for serum sodium.

Abbreviations: ACLF, acute‐on‐chronic liver failure; ALD, alcohol‐associated liver disease; HR, hazard ratio; ICU, intensive care unit.

Rebleeding occurred in 19 patients (8.3%), with 7 cases (3.0%) classified as early rebleeding (within 5 days) and 12 cases (5.3%) as late rebleeding. Among them, 7 patients died, resulting in a mortality rate of 36.8% within the rebleeding subgroup. Table 4 presents the bivariate Cox regression analysis, identifying associations between independent variables and the incidence of rebleeding. Table 5 summarizes the multivariate analysis, which revealed that orotracheal intubation was independently associated with an increased risk of rebleeding, whereas ICU transfer was associated with a reduced risk.

**TABLE 4:** Bivariate Cox regression analysis showing associations between independent variables and rebleeding.

Variable	HR	95% confidence interval		*p*
Demographics				
Sex	0.920	0.318	2.665	0.878
Age	1.006	0.965	1.048	0.793
Causes of cirrhosis				
ALD	0.966	0.353	2.645	0.947
MASLD	1.050	0.288	3.823	0.941
MetALD	1.119	0.145	8.625	0.914
Cryptogenic cirrhosis	0.601	0.078	4.648	0.625
Past medical history				
Previous upper gastrointestinal bleeding	2.817	1.039	7.635	0.042
HCC	2.541	0.317	20.362	0.380
Diabetes	1.246	0.451	3.441	0.671
Manifestations of gastrointestinal bleeding				
Hematemesis	0.638	0.217	1.882	0.416
Melena	2.763	0.842	9.064	0.094
Hematemesis and melena	1.299	0.474	3.560	0.611
New‐onset anemia	0.048	0	267200000	0.791
Cirrhosis complications at admission				
Ascites	2.369	0.854	6.574	0.098
Hepatic encephalopathy	1.126	0.799	1.586	0.498
Laboratory values				
Hemoglobin	0.893	0.722	1.104	0.296
Hematocrit	0.963	0.895	1.037	0.321
White blood cells	1.000	1.000	1.000	0.703
Neutrophils	1.000	1.000	1.000	0.526
Lymphocytes	1.000	0.999	1.001	0.539
Platelets	1.000	1.000	1.000	0.283
Sodium	0.969	0.887	1.057	0.476
Serum total proteins	0.709	0.463	1.084	0.112
Blood urea nitrogen	1.004	0.985	1.024	0.652
Liver disease severity scores				
CAGIB	1.123	0.797	1.582	0.508
MELD	0.974	0.907	1.046	0.472
MELD‐sodium	0.992	0.940	1.047	0.776
Child‐Pugh	1.130	0.887	1.440	0.321
ACLF	1.356	0.921	1.997	0.123
In‐hospital events				
Balloon tamponade	2.782	0.913	8.478	0.072
Intubation/mechanical ventilation	3.022	1.037	8.811	0.043
ICU transference	2.375	0.829	6.800	0.107
Vasoconstrictor	4.954	0.112	219.8	0.408
Lactulose	0.255	0.058	1.128	0.072
Proton pump inhibitors	0.474	0.054	4.183	0.502
HCC diagnosed after admission	1.892	0.235	15.255	0.549
Transfusions	2.493	0.855	7.269	0.094
Units of packed red blood cells	1.556	1.064	2.276	0.023
Units of fresh frozen plasma	1.075	0.819	1.412	0.603
Units of platelets	0.049	0	53140000000000000	0.887
Units of cryoprecipitate	0.667	0.002	186	0.888
Endoscopic assessment and therapy				
Esophageal varices	0.567	0.118	2.709	0.477
Gastric varices	0.035	0	8.585	0.233
Variceal bleeding	3.036	0.400	23.037	0.283
Peptic ulcer bleeding	0.937	0.122	7.179	0.950
Other causes of bleeding	0.338	0.045	2.565	0.294
Band ligation	1.687	0.377	7.542	0.494
Cyanoacrylate injection	0.041	0	37.143	0.358
Peptic ulcer treatment	4.388	0.525	36.670	0.172

*Note:* CAGIB, cirrhosis acute gastrointestinal bleeding; HCC, hepatocellular carcinoma; MELD‐sodium, MELD adjusted for serum sodium.

Abbreviations: ACLF, acute‐on‐chronic liver failure; ALD, alcohol‐associated liver disease; HR, hazard ratio; ICU, intensive care unit; MELD, model for end‐stage liver disease.

**TABLE 5:** Multivariate Cox regression model to evaluate rebleeding.

Variable	HR	95% confidence interval		*p*
Manifestations of gastrointestinal bleeding				
Melena	1.35	0.20	9.30	0.763
Past medical history				
Previous upper gastrointestinal bleeding	3.63	0.73	18.15	0.116
Cirrhosis complications at admission				
Ascites	2.45	0.51	11.71	0.263
Laboratory values				
Serum total proteins	0.84	0.40	1.74	0.635
Liver disease severity scores				
ACLF	0.69	0.28	1.70	0.425
In‐hospital events				
Balloon tamponade	0.63	0.08	4.76	0.654
Intubation/mechanical ventilation	88.03	2.64	2930.91	0.012
Lactulose	0.20	0.03	1.30	0.092
ICU transference	0.04	0.00	0.93	0.045
Transfusions	1.51	0.07	30.58	0.789
Units of packed red blood cells	1.37	0.45	4.14	0.580
Endoscopic assessment and therapy				
Peptic ulcer treatment	4.28	0.26	71.27	0.311

Abbreviations: ACLF, acute‐on‐chronic liver failure; HR, hazard ratio; ICU, intensive care unit.

Infections were documented in 55 patients (24.1%), with the majority occurring during the first week of hospitalization. Ten patients (4.4%) developed infections after the first week, and nine individuals presented with infections at multiple anatomical sites. Pneumonia was the most frequent infection, observed in 28 patients (12.3%), followed by urinary tract infections in 17 (7.5%), spontaneous bacterial peritonitis (SBP) in 9 (3.9%), and infections at other sites (including cellulitis, osteomyelitis, and bloodstream infections) in 10 patients (5.1%).

Microbiological cultures identified infective agents in 26 patients (11.4%). Staphylococcus species were the most commonly isolated pathogens, found in 12 cases: *S*. *epidermidis* (*n* = 6), *S. hominis* (*n* = 4), *S. aureus* (*n* = 1), and *S. capitis* (*n* = 1). *Klebsiella pneumoniae* was isolated in 8 patients and *Klebsiella oxytoca* in 2. Candida species were identified in 5 patients, with *Candida albicans* present in 4 of them. *Escherichia coli* was found in 4 cases, *Enterococcus faecalis* in 2, and *Pseudomonas* species in 2. Other organisms isolated in single cases included *Micrococcus luteus*, *Stenotrophomonas maltophilia*, and *Citrobacter* species. Table 6 presents the results of the Poisson linear regression analysis, showing bivariate associations between independent variables and the incidence of infections. Table 7 summarizes the multivariate analysis, which identified prolonged hospitalization and elevated Child‐Pugh scores as independent predictors of infection.

**TABLE 6:** Poisson linear regression analysis showing bivariate associations between independent variables and infections.

Variable	Poisson response	95% confidence interval		*p*
Demographics				
Sex	1.298	0.723	2.332	0.382
Age	1.021	0.999	1.042	0.056
Causes of cirrhosis				
ALD	1.000			
MASLD	0.788	0.401	1.547	0.489
MetALD	1.333	0.402	4.428	0.639
Hepatitis C	1.200	0.361	3.985	0.766
Hepatitis B	0.500	0.068	3.696	0.497
Cryptogenic cirrhosis	1.000	0.347	2.882	1.000
Other causes of liver cirrhosis	1.273	0.548	2.954	0.575
Past medical history				
Previous upper gastrointestinal bleeding	0.888	0.490	1.609	0.695
Diabetes	0.981	0.577	1.667	0.943
Hematemesis	1.000			
Manifestations of gastrointestinal bleeding				
Melena	0.628	0.287	1.375	0.245
Hematemesis and melena	0.602	0.307	1.180	0.140
Hematochezia	0.929	0.306	2.822	0.897
New‐onset anemia	0.315	0.041	2.422	0.267
Cirrhosis complications at admission				
Ascites	1.066	0.401	2.838	0.898
Hepatic encephalopathy	0.641	0.082	5.016	0.671
Laboratory values				
Hemoglobin	1.365	0.566	3.292	0.489
Hematocrit	1.015	0.732	1.406	0.929
Red blood cells	0.438	0.160	1.197	0.107
White blood cells	1.000	0.999	1.000	0.562
Neutrophils	1.000	1.000	1.001	0.486
Lymphocytes	1.000	1.000	1.001	0.710
Platelets	1.000	1.000	1.000	0.772
Sodium	0.996	0.945	1.049	0.870
Creatinine	1.497	0.253	8.862	0.657
Serum total proteins	0.789	0.594	1.047	0.101
Liver disease severity scores				
Child‐Pugh	1.250	1.062	1.471	0.007
CAGIB	1.121	0.815	1.542	0.483
MELD	0.994	0.923	1.071	0.879
ACLF	3.345	1.712	6.537	< 0.001
In‐hospital events				
No abdominal imaging	1.000			
Ultrasonography	0.859	0.372	1.984	0.721
Computed tomography	2.060	1.122	3.782	0.020
Hepatocellular carcinoma	2.060	2.060	2.060	2.060
Balloon tamponade	3.502	1.356	9.046	0.010
Intubation/mechanical ventilation	3.032	0.805	11.421	0.101
Intubation due to drowsiness	1.000			
Intubation due to hemodynamic shock	1.523	0.199	11.638	0.685
No antibiotic	1.000			
Ceftriaxone	1.369	0.165	11.349	0.771
Cefepime	3.110	0.254	38.060	0.375
Two or more antibiotics	5.477	0.621	48.273	0.126
Meropenem	8.786	0.692	111.617	0.094
Lactulose	1.358	0.786	2.347	0.273
Proton pump inhibitors	2.761	0.951	8.013	0.062
Transfusions	0.766	0.313	1.874	0.559
Units of packed red blood cells	1.041	0.706	1.534	0.840
ICU transference	1.295	0.345	4.859	0.701
Hospitalization length (days)	1.075	1.052	1.098	< 0.001
Endoscopic assessment and therapy				
Esophageal varices	0.510	0.179	1.451	0.207
Gastric varices	1.264	0.622	2.568	0.516
Variceal bleeding	1.955	0.592	6.451	0.271
Peptic ulcer bleeding	2.137	0.713	6.407	0.175
Bleeding due to PHG	0.736	0.158	3.424	0.696
Bleeding due to esophagitis	1.284	0.291	5.665	0.741
Other causes of bleeding	1.411	0.449	4.433	0.555
Band ligation	1.078	0.520	2.234	0.840
Cyanoacrylate injection	1.803	0.658	4.937	0.251
Peptic ulcer treatment	1.461	0.412	5.181	0.558
Other endoscopic treatment	1.426	0.547	3.721	0.468

*Note:* CAGIB, cirrhosis acute gastrointestinal bleeding; MASLD, metabolic dysfunction–associated steatotic liver disease; MELD, model for end‐stage liver disease; MetALD, metabolic dysfunction and alcohol‐associated liver disease.

Abbreviations: ACLF, acute‐on‐chronic liver failure; ALD, alcohol‐associated liver disease; ICU, intensive care unit.

**TABLE 7:** Poisson multiple linear regression analysis showing associations between independent variables and infections.

Variable	Exp (B)	95% Wald confidence interval for exp (B)		*p*
Demographics				
Age	1.006	0.983	1.030	0.611
Manifestations of gastrointestinal bleeding				
Hematemesis and melena	0.523	0.238	1.149	0.106
Laboratory values				
Red blood cells	1.243	0.802	1.927	0.331
Serum total proteins	0.797	0.571	1.112	0.181
Liver disease severity scores				
Child‐Pugh	1.159	1.009	1.333	0.037
ACLF	2.923	0.701	12.186	0.141
In‐hospital events				
Computed tomography	1.008	0.497	2.045	0.982
Balloon tamponade	0.523	0.159	1.717	0.285
Intubation/mechanical ventilation	1.274	0.317	5.124	0.733
Two or more antibiotics	1.310	0.140	12.251	0.813
Meropenem	16.138	0.747	348.437	0.076
Proton pump inhibitors	1.561	0.452	5.394	0.481
Hospitalization length (days)	1.064	1.029	1.100	< 0.001
Endoscopic assessment and therapy				
Peptic ulcer bleeding	1.672	0.621	4.506	0.309

Abbreviation: ACLF, acute‐on‐chronic liver failure.

## 4. Discussion

One of the key findings of this study was the clinical evidence supporting ICU management for patients with cirrhosis and UGIB. Although the association between ICU transfer and reduced mortality did not reach conventional statistical significance (*p* = 0.070), a similar trend was previously reported in a retrospective study [12]. Furthermore, the significant association between ICU transfer and lower risk of rebleeding reinforces the importance of early ICU management in this population. These findings align with prior observations that delays in ICU referral are linked to increased rates of rebleeding and mortality [12].

Although the difference in survival rates between patients with alcohol abstinence and those who continued drinking did not reach statistical significance in this study—likely due to high variability—alcohol abstinence remains a clinically relevant factor in ALD management. Abstinence has been shown to reduce portal hypertension, thereby potentially mitigating blood loss during variceal bleeding episodes [17]. Unfortunately, few studies investigating ALD and UGIB include detailed information on alcohol abstinence, and most do not stratify patients based on this variable. It underscores the need for future research to incorporate abstinence status as a key prognostic factor.

The increased mortality observed among patients receiving higher volumes of PRBCs was also relevant. Previous research has demonstrated that a restrictive transfusion strategy, targeting serum hemoglobin levels between 7 and 9 g/dL, results in improved survival compared to a liberal strategy aiming for levels between 9 and 11 g/dL. This benefit is likely attributable to a reduction in rebleeding rates, which decreased from 16% to 10% under the restrictive approach. [18]. Of note, five patients in our cohort (2.1%) received FFP, although current guidelines generally do not recommend its use for managing coagulopathy in cirrhosis [19]. Nonetheless, FFP may be indicated in specific situations, such as disseminated intravascular coagulation or in the context of severe bleeding with abnormal coagulation parameters. In our study, FFP was administered exclusively to patients presenting with massive bleeding and elevated INR values. Unfortunately, despite receiving FFP, all five of these patients died.

Despite the lower mortality and rebleeding rates observed in this study compared to others [9, 12, 20, 21], both outcomes were significantly influenced by orotracheal intubation. A previous study involving 113 patients with cirrhosis who underwent mechanical ventilation found that the primary reason for intubation was altered mental status, which was also the case for our patients. Furthermore, the authors reported that 53% of their patients died, and 51% were extubated; however, only 36% maintained patent airways for more than 72 h [22]. Notably, three of our intubated patients remained in medical wards awaiting transfer to the ICU, which likely worsened their outcomes following mechanical ventilation. Given that intubation emerged as the most significant variable affecting mortality and rebleeding in this study, it is crucial that research on variceal bleeding not be confined to assessing risk factors at the time of admission. Failing to do so may overlook important variables, such as mechanical ventilation, infections, and organ failure.

Among the 19 cases of rebleeding, no significant differences in hemoglobin levels, MELD scores, or endoscopic treatments were observed between the 10 patients admitted to the ICU and the 9 patients managed in medical wards. We hypothesize that the latter group may have been in a similarly severe condition but received inadequate fluid resuscitation. Under this assumption, more appropriate and closely monitored fluid management in the ICU could explain the reduction in rebleeding risk associated with ICU accessibility.

Patients with cirrhosis are at a heightened risk of developing infections, a situation exacerbated in those with ALD due to their impaired immune response. This impairment is attributed to a reduced quantity of mucosal‐associated invariant T cells in the bloodstream, which compromises their antibacterial defense [23]. In our study, we found that both the Child‐Pugh score and the length of hospitalization were directly associated with infection rates, indicating that advanced liver disease is a significant risk factor. Furthermore, the incidence of infections prolongs hospitalization, likely due to clinical deterioration and the necessity of awaiting intravenous antibiotic administration.

In the present study, pneumonia was the most frequent site of infection, with *Klebsiella pneumoniae* and *Staphylococcus epidermidis* being the predominant pathogens. While the majority of studies on infections in cirrhotic patients focus on SBP, the most commonly reported organisms in that context include *E*. *coli*, *S*. *epidermidis*, and *E*. *faecium*. However, a wide range of other pathogens may also be involved, and nearly half of the bacterial isolates reported are multi–drug‐resistant, underscoring the need for careful antibiotic stewardship and early microbiological surveillance [24]. A previous study investigating *K. pneumoniae* infections in patients with cirrhosis showed that infections caused by this pathogen are relatively common, affecting approximately 19.4% of hospitalized individuals. Of note, UGIB was identified as an independent risk factor for *K. pneumoniae* infection [25].

Current clinical guidelines recommend antibiotic prophylaxis for patients with cirrhosis and UGIB. Accordingly, 219 patients (96%) in our cohort received prophylactic antibiotics. However, a recent systematic review failed to identify clinical evidence supporting this recommendation, highlighting the need for further prospective studies to clarify the role of prophylaxis in preventing infections following UGIB in cirrhotic patients [26]. For instance, the role of antibiotic prophylaxis in fungal infections is yet to be determined.

This study has several limitations. It was observational, nonrandomized, and conducted at a single center. Additionally, some statistical associations might have differed with a larger sample size. Nonetheless, the study presents important strengths. The statistical analyses were guided exclusively by mathematical criteria, with variables selected for multivariate models based on predefined thresholds in bivariate analysis. Furthermore, the prospective design allowed for real‐time inclusion and follow‐up of patients, enabling direct observation of exposures and clinical outcomes.

## 5. Conclusions

The findings of this prospective study suggest that access to ICU management may confer protection against rebleeding. Orotracheal intubation emerged as one of the strongest independent risk factors for both mortality and rebleeding in this population. Additionally, ALD was associated with lower mortality rates, whereas the volume of PRBCs transfused correlated with increased risk of death. Infections were independently associated with prolonged hospitalization and elevated Child‐Pugh scores [5, 6].

## Author Contributions

Mariana Barros Marcondes: methodology and investigation; formal analysis; data curation; writing–original draft; and writing–review and editing. Clara Fantinelli Moreno: methodology; investigation; formal analysis; data curation; and writing–original draft. Cíntia Mitsue Pereira Suzuki: data curation; investigation; and writing–original draft. Maxwell Antonio Garcia Rodrigues: data curation; investigation; and writing–original draft. Alecsandro Moreira: data curation; investigation; and writing–original draft. Xingshun Qi: conceptualization; methodology; writing–original draft; writing–review and editing; supervision; and project administration. Fernando Gomes Romeiro: conceptualization; methodology; investigation; formal analysis; data curation; writing–original draft; writing–review and editing; supervision; project administration; and funding acquisition.

## Funding

Fernando Gomes Romeiro received support from the Conselho Nacional de Desenvolvimento Científico e Tecnológico (CNPq), Grant No. 304131/2024‐5. This work was also supported by the São Paulo Research Foundation (FAPESP) through Grant No. 2023/14281‐3.

## Conflicts of Interest

The authors declare no conflicts of interest.

## Data Availability

The data that support the findings of this study are available upon request from the corresponding author. The data are not publicly available due to privacy or ethical restrictions.
